# Love in Quarantine: Sexting, Stress, and Coping During the COVID-19 Lockdown

**DOI:** 10.1007/s13178-021-00645-z

**Published:** 2021-09-23

**Authors:** Dora Bianchi, Roberto Baiocco, Antonia Lonigro, Sara Pompili, Marta Zammuto, Daniele Di Tata, Mara Morelli, Antonio Chirumbolo, Anna Di Norcia, Eleonora Cannoni, Emiddia Longobardi, Fiorenzo Laghi

**Affiliations:** 1 Department of Developmental and Social Psychology, Sapienza University of Rome, Rome, Italy; 2 Department of Human Sciences, European University of Rome, Rome, Italy; 3 Department of Dynamic and Clinical Psychology, and Health Studies, Sapienza University of Rome, Rome, Italy; 4 Department of Psychology, Sapienza University of Rome, Rome, Italy

**Keywords:** Sexting, Coping, Pandemic-related stress, Emerging adults, COVID-19 lockdown

## Abstract

**Introduction:**

This study investigated the relationships of pandemic-related stress and coping strategies with different kinds of sexting (i.e., experimental, risky, and emotional) during the COVID-19 lockdown in the Italian context.

**Methods:**

A sample of 1929 emerging adults (*M*_*age*_ = 24.17, *SD*_*age*_ = 2.75; 71.6% girls) completed an online survey about their sexting behaviors during the national lockdown in Italy. Data were gathered in April/May 2020, from 6th to 11th week of home confinement due to COVID-19 pandemic. Hierarchical regression and mediation analyses were performed.

**Results:**

Pandemic-related stress directly predicted only risky and emotional sexting. Experimental and emotional sexting were positively predicted by social support, and negatively predicted by turning to religion. Risky and emotional sexting were positively predicted by avoidance, and negatively predicted by problem solving. Adaptive coping (i.e., social support) mediated the relationships from pandemic-related stress to both experimental and emotional sexting. Maladaptive coping (i.e., avoidance and problem solving) mediated the relationships from pandemic related-stress to risky and emotional sexting.

**Conclusions:**

Sexting was a coping tool during COVID-19 lockdown, showing both adaptive and maladaptive facets.

**Policy Implications:**

Findings suggest new directions for implementing programs of sexual education and safer Internet use targeted to young people.

## Introduction

With the outbreak of the coronavirus disease 2019 (COVID-19) pandemic, the Italian government imposed a national lockdown from March to May 2020 to contrast the spread of infection, with the ban on leaving home except for essential needs. Non-cohabiting people were strictly forbidden to meet each other and physical contacts became extremely dangerous, while social distancing and home-confinement were indispensable measures to avoid the contagion. Emerging adults, less exposed to the health-risks of COVID-19 infection, surprisingly turned out to be the most sensitive age group for pandemic-related stress (Carli et al., 2020; Kujawa et al., 2020; Laghi et al., 2021). The instability typical of this age, as a phase of incomplete definition of adult identity (Arnett et al., 2014), was indeed exacerbated by the pandemic condition. In Italy, many emerging adults suffered job suspension or dismissal, or had to leave their temporaneous living arrangements to spent quarantine with their families, so giving up much of their recently obtained independence (Parola, 2020). The pandemic also impacted on their social and intimate life, that during this developmental stage is mostly centered outside family, with friends, non-cohabiting romantic partners, or short-term mating (Collins & van Dulmen, 2006). Due to the lockdown’s restrictions in Italy, non-cohabiting partners found themselves suddenly divided, and their attempts to meet each other were systematically prevented by police surveillance in the streets. Therefore, the lockdown disrupted emerging adults’ intimate relationships imposing sexual abstinence and physical distancing (Döring, 2020).

In this condition, many youths turned to the online dimension for maintaining their social and romantic relationships (Lindberg et al., 2020), and it is conceivable that new technologies have been of support also for sexual contacts. Scholars anticipated an increase in different online sexual activities during lockdown, including sexting (Alpalhão & Filipe, 2020; Lindberg et al., 2020), which is currently considered a new normality among young people (Döring, 2014). In the present study, we specifically focuses on three facets of sexting: (1) experimental, in which the sexual contents are shared within a relationship of trust (Wolak et al., 2012); (2) risky, implying more risk-taking (Morelli et al., 2020, 2021); and (3) emotional, when sexting is enacted to reduce negative feelings, resembling other dysfunctional behaviors (Bianchi et al., 2019a). There is still few research about sexting behaviors among emerging adults during COVID-19 lockdown, and no data are yet available about the Italian population. However, sexting might have helped youths to fulfill sexual desires and to maintain intimate relationships, while facing forced social isolation. Thus, within the theoretical framework of the transactional stress and coping model (Lazarus & Folkman, 1984), the present study aims to understand how emerging adults engaged in different sexting behaviors to cope with pandemic-related difficulties during the COVID-19 lockdown in Italy.

### Sexting Behaviors in Emerging Adults

The term “sexting” refers to self-made sexually suggestive messages, photos, and videos, shared by new technology devices (Chalfen, 2009). Within this general definition, we can found very different forms of sexting (reviews by Mori et al., 2019, 2020), ranging from safer and intimate communications, in which sexual contents are shared with a partner to explore sexuality in a relationship of trust (i.e., experimental sexting; Wolak et al., 2012), to progressively more risky behaviors. In its high-risk forms, sexting may co-occur with other dangerous behaviors, such as use of substances that reduce perception of risk and consensus, and/or engaging in sexting with strangers, increasing the probability that own sexual contents would be later disseminated online (i.e., risky sexting; Morelli et al., 2020, 2021). Higher likelihood to engage in sexting has been found indeed for young people who make alcohol and drugs use (Mori et al., 2019), and for youths who meet strangers online (Crimmins & Seigfried-Spellar, 2014). Recent studies (Bianchi et al., 2019a; Yoder et al. 2018) also shed light on a facet more akin to Internet addiction: the use of sexting to escape negative feelings, in absence of more effective emotion regulation skills (i.e., emotional sexting). Yoder et al. (2018) uncovered emotional motivations for sexting, related to anger, loneliness, and attention seeking, which correlate with more risky behaviors. Emotion dysregulation and emotional problems are proven to predict sexting, as well as other risky sexual behaviors, in young people (Ševčíková, 2016).

Sexting behaviors progressively increase during adolescence reaching a peak in emerging adulthood (Bianchi et al., 2019b; Mori et al., 2020). Emerging adults are still in an exploratory phase regarding their sexuality and intimate relationships (Morgan, 2013), often fluctuating between committed relationships and short-term mating (Shulman & Connolly, 2013). Therefore, they can be more open to explore new sexual behaviors including sexting (Mori et al., 2020), with both stable partners and short-term mates (Brodie et al., 2019). Experimental sexting in emerging adulthood has a prevalence from 47 to 77% (Brodie et al., 2019; Morelli et al., 2021; Mori et al., 2020), but also risky sexting (about 43%; Morelli et al., 2020, 2021) and emotional sexting (30%; Bianchi et al., 2019a) are very frequent. Recent research (Bianchi et al., 2021; Morelli et al., 2020, 2021) has shown that experimental sexting is equally spread across different biological sex and sexual orientation groups, whereas risky forms of sexting are more frequent among boys, who are generally more prone to at-risk behaviors (Korn & Bonny-Noach, 2018), and among sexual minority youths, who are more vulnerable to risky sexual behaviors (Nappa et al., 2021). Despite their frequency, these different kinds of sexting are rarely explored in research, because most studies only assess overall sexting behaviors. However, the new condition of COVID-19 lockdown, which suddenly increased feelings of loneliness, frustration, and fear, might have provided several motives to young people to engage in experimental, risky, and emotional sexting.

### Sexting Behaviors and COVID-19 pandemic

The impact of COVID-19 lockdown on sexual behaviors is currently an emerging research topic (reviews by Döring, 2020; Eleuteri & Terzitta, 2021). When the pandemic began, public health institutions advocated the need to avoid sexual contacts, suggesting online sexual activities as a safer alternative to prevent contagion ( e.g. International Society for the Study of Women’s Sexual Health, 2020). Thus, sexting and other virtual sexual behaviors were encouraged during lockdown, even if their risks for psychological well-being remained a major concern (Alpalhão & Filipe, 2020).

Moreover, home-confinement may have encouraged sexting among emerging adults in different ways. On one hand, not-cohabiting couples were suddenly forced to live at-distance (Wijayanti, 2021), and sexting might have been a good alternative to maintain intimacy and fulfill sexual desire. On the other hand, many emerging adults were used to have casual sexual encounters or noncommitted sexual relationships, that during pandemic were largely forbidden (Wignall et al., 2021): Also in this case, sexting and online sexual activities could have been a safer way to satisfy sexual desire. Overall, many youths were home-confined with their families, a condition that drastically reduced their independence (Hall & Zygmunt, 2021), and online sexual communication became one of the few available means to express their sexuality.

Accordingly, a general increase in online sexual behaviors, including sexting, has been registered during COVID-19 lockdown (Ballester‑Arnal et al., 2020; Gabster et al., 2021; Lehmiller et al., 2020). During national quarantine, online sexual activities were reported in percentages between 28% and 38% (Ballester‑Arnal et al., 2020; Gabster et al., 2021), and their increase was associated to a reduction of traditional casual sex (Gabster et al., 2021). The 15% of emerging adults started to use sexting during quarantine, reporting as a consequence more satisfaction for their sexual life (Lehmiller et al., 2020). An increase in sexting during lockdown was also specifically registered among sexual minorities (Nelson et al., 2020). Lesbian, gay, and bisexual (LGB) youths may have been more at risk for distress during lockdown, because they were home-confined with their families, who could be unwelcoming toward their sexual orientation (Woznicki et al., 2020). Therefore, these youths might have turned to the online dimension to communicate with friends, to find emotional support, and also to express their sexuality during home-confinement (Eleuteri & Terzitta, 2021; Woznicki et al., 2020).

While the increase in online sexual activities during COVID-19 pandemic has been relatively confirmed, the role they have had for psychological well-being is still relatively unexplored. To date, there is evidence that sexting was more reported by people who suffered higher pandemic-related stress (Lehmiller et al., 2020). During pandemic, online sexual activities shared with the partner were protective for sexuality, intimacy and relational well-being, while conversely solitary online sexual behaviors were associated with more psychological distress (Rodrigues & Martins, 2020). This initial evidence suggests the importance to further investigate the relationship between sexting and pandemic-related stress during COVID-19 lockdown.

Pre-pandemic studies indicated that emerging adults used sexting to flirt with actual or potential partners, to fuel passion and intimacy in established relationships, and to maintain sexual desire in long-distance relationships (Van Ouytsel et al., 2020). Out of a couple relationship, sexting was moved by desires of thrill and excitement (Van Ouytsel et al., 2017), and frequently associated with other risk behaviors (Dir et al., 2013). Therefore, sexting during COVID-19 lockdown might have been used as a coping tool, to reduce negative feelings associated with pandemic, and to maintain sexual contacts during home confinement.

### Coping with Pandemic-Related Stress and Online Behaviors During Lockdown

The COVID-19 outbreak had a negative impact on psychological well-being of emerging adults (Birditt et al., 2021; Kujawa et al., 2020; Laghi et al., 2021), entailing primary stressors (fears of contagion and its consequences for own and others’ health), and secondary stressors (worrying about social isolation, financial and job insecurity, and resource scarcity; Zheng et al., 2021). These stressors have increased general levels of depression, anxiety, and perceived loneliness in youths (Birditt et al., 2021; Kujawa et al., 2020). High rates of pandemic-related stress (41.6%), depression (12.4%), and anxiety (17.6%) were also found in Italian population (Fiorillo et al., 2020).

Recent research has uncovered different coping mechanisms adopted by young people to face these pandemic-related stressors (Park et al., 2020). According to the transactional theory of stress and coping (Lazarus & Folkman, 1984), stress is the exposure to environmental stimuli individually appraised as harmful, threatening, or extremely challenging—such as the abovementioned pandemic conditions. The perceived distress activates coping processes, aimed to re-establish emotional well-being. Coping is the cognitive and behavioral attempt to manage environmental stressors (Lazarus & Folkman, 1984), and it may be problem-focused (i.e., oriented to solve the conflict between individual and environment), or emotion-focused (i.e., oriented to manage emotional states evoked by the situation; Lazarus & Folkman, 1984). Moreover, coping can be approaching (i.e., oriented toward the source of stress and related emotions and thoughts) or avoidant (i.e., oriented away from the stressor and related emotions and thoughts; Compas et al., 2001). During the COVID-19 lockdown, the most frequent coping strategies adopted to face pandemic-related stress have been the following: emotion-focused, such as looking for social and emotional support, turning to religion, and positive thinking; problem-focused, such as adopting behaviors to reduce risks for contagion; and avoidance-oriented, related to denial of risk situation, searching for distraction, and making substance abuse (Park et al. 2020; Pirutinsky et al., 2020). Overall, a coping strategy is considered adaptive or maladaptive according to interactions between individual and environmental factors (Lazarus & Folkman, 1984). Problem-focused coping may be effective for controllable sources of distress, whereas emotion-focused coping is more adaptive in presence of uncontrollable situations (Lazarus & Folkman, 1984). During COVID-19 pandemic, adaptive coping strategies which effectively reduced pandemic-related stress, were mostly emotion-focused, such as looking for social support, positive thinking, and looking for distraction (Park et al., 2020; Rettie & Daniels, 2020), and only in minor part problem-focused, such as prevention behaviors for health and financial conditions (Wang et al., 2020). Conversely avoidant coping, related to substance abuse and denial of pandemic risks, was considered maladaptive and increased symptoms of psychological distress (Rettie & Daniels, 2020).

Young people have also used new media to cope with pandemic-related stress (Garfin, 2020). The coping function of online behaviors is widely acknowledged (review by Wolfers & Schneider, 2020) and, specifically during home-confinement, new technologies might have served for problem-focused coping (i.e., retrieving information about contagion and prevention) and for emotion-focused coping (i.e., maintaining social contacts, looking for emotional support; Garfin, 2020). Also avoidant-coping might have been implemented, because spending time on Internet can be a way to escape from emotional troubles and negative affect (Kardefelt-Winther, 2014). Problematic Internet use, as well as risky online behaviors, are characterized by a maladaptive use of the online dimension to escape negative feelings, in place of more effective coping strategies (Kardefelt-Winther, 2014; Melodia et al., 2020). Research suggests that emerging adults have used online behaviors in different ways to cope with COVID-19 lockdown, but no studies have yet investigated this specific use of sexting. However, also sexting behaviors might have been used as a result of coping strategies to face pandemic-related stress.

## The Current Study

In line with the model of stress and coping theorized by Lazarus and Folkman (1984), the present study investigated the relationships between pandemic-related stress, coping strategies, and different facets of sexting—i.e., experimental, risky, emotional sexting—in emerging adults during the COVID-19 lockdown in Italy. Recent research addressed an increase in sexting during COVID-19 lockdown in different countries (e.g. Lehmiller et al., 2020), however no research has yet been conducted in the Italian context. Moreover, the role of different sexting behaviors as possible responses to pandemic-related difficulties is still unexplored. Thus, we aimed to answer the following research questions:

Q1: Was there any difference in experimental, risky, and emotional sexting, in varying relationship conditions—being at long-distance, non-distance, or not-in couple—during the COVID-19 lockdown in Italy? Pre-pandemic studies only suggested that sexting is more frequent in long-distance relationships (Van Ouytsel et al., 2020), while it is conceived as a form of risk-taking outside a couple relationship (Van Ouytsel et al., 2017).

Q2: Controlling for individual and relationship differences, did pandemic-related stress and coping strategies predict engagement in experimental, risky, and emotional sexting during COVID-19 lockdown? Recent literature has suggested indeed that online behaviors may be used to cope with distressing situations and emotions (Wolfers & Schneider, 2020).

Q3: Did coping strategies differently mediate the relationship between pandemic-related stress and each sexting behavior? According with studies on the coping role of online behaviors during pandemic (Garfin, 2020), we hypothesized that experimental sexting would be associated to pandemic related-stress via emotion-oriented coping, as youths may look for emotional support by their sexting partners within a relationship of trust (H1). Conversely, in line with research on problematic Internet use (Kardefelt-Winther, 2014), we hypothesized that risky and emotional sexting would be associated to pandemic-related stress via avoidant-coping, since they could function as means of distraction from negative feelings and thoughts associated with pandemic (H2).

## Method

### Participants and Procedure

This study enrolled 1929 emerging adults from 18 to 29 years old (*M*_*age*_ = 24.17, *SD*_*age*_ = 2.75; 71.6% girls). As inclusion criterion, all participants lived in Italy during the COVID-19 national lockdown. Most of them (96.7%) were Italian, while a small percentage (3.3%) were immigrants. As regard their sexual orientation, 91.8% were exclusively heterosexual, while 8.2% were lesbian, gay, bisexual, or with other sexual orientations (LGB+). About their relationship status, 1149 participants (59.6%) were currently involved in a dating relationship (vs. 780 who were not in couple), with 817 (42.4%) reporting a long-distance relationship with their dating partner during the current lockdown (vs. 322 non-distance relationships), while only 254 (13.2%) reported a long-distance relationship before the lockdown (vs. 895 non-distance). Data were gathered from the 6th to the 11th week of home-confinement (April/May, 2020), during the national lockdown imposed in Italy for contrasting the spread on COVID-19 contagion. Participants were contacted online via a snowball sampling method, sharing the link of the online survey through the University Web site. Initially, 2566 emerging adults matching the inclusion criteria were invited, and an informed consent was presented in the first page of the survey, ensuring that the participation was voluntary and anonymous. Only 1929 participants accepted to participate and correctly completed the survey, resulting in a response rate of 75.2%. Power analyses were run using the G*Power software program, version 3.1. Considering the conventional levels of 80% power and 0.05 alpha significance (Cohen, 1988), the a-priori power analysis suggested a required minimum sample size of 395 participants to detect small effects (Cohen’s* d* = .20). The subsequent post-hoc power analysis indicated that the actual sample size was 99% power to detect small effect sizes.

### Measures

#### Individual Information

Participants reported their biological sex, age, and nationality.

#### Sexual Orientation

Participants reported their sexual orientation as: heterosexual; lesbian; gay; bisexual; other sexual orientations. Following a procedure suggested in previous studies (e.g., Pistella et al., 2019), sexual orientation was then dummy coded for the purposes of our study (0 = heterosexual; 1 = LGB+).

#### Relationship Status

Participants reported if they were currently involved in a dating relationship, defined as “spending time with a person you love, like, or have a crush on” (definition adapted by Connolly et al., 2004), and their relationship status was dummy coded (0 = not in couple; 1 = in couple). Participants also reported whether they were in a long-distance relationship with their dating partner (0 = no, 1 = yes) during the current lockdown. According to Guldner (1996), long-distance relationships were described as “your partner lives far enough away from you that it is impossible or very difficult to see each other every day.” Participants were also classified in three groups according to their relationship status during lockdown (not in couple; non-distance relationship; long-distance relationship).

#### Pandemic-Related Stress

The stress suffered for the overall situation of pandemic was assessed by one item (“Please, indicate how stressful is for you the emergency situation related to the spread of COVID-19 pandemic”), with answers rated on a 5-point Likert-type scale from 1 (*not at all*) to 5 (*very much*). This measure closely resembled other single-item measures of pandemic-related stress adopted in recent studies (e.g., Birditt et al., 2021), and it is in line with the item 7 of the Coronavirus Impact Scale (Stoddard & Kaufman, 2020), that was designed to investigate stress due to COVID-19 pandemic.

#### Coping Strategies

The 60-item Italian version of the Coping Orientation to the Problems Experienced scale (COPE-NVI, Sica et al., 2008) evaluated five coping strategies in response to stressful situations. Items were rated on a 4-point Likert-type scale, from 1 (*I usually don’t do this at all*) to 4 (*I usually do this a lot*). The five coping dimensions were: social support, i.e., looking for comprehension, information, and emotional support (12 items; sample item: “I try to get advice from someone about what to do”; Cronbach’s alpha of .90); positive attitude, i.e., tendency to accept and positively redefine the situations (12 items; sample item: “I look for something good in what is happening”; Cronbach’s alpha of .78); avoidance strategies, i.e., behaviors aimed to avoid emotional awareness of situations (16 items; sample item: “I refuse to believe that it has happened”; Cronbach’s alpha of .79); problem solving, i.e., planning and problem solving strategies (12 items; sample item: “I focus on dealing with this problem, and if necessary let other things slide a little”; Cronbach’s alpha of .85); turning to religion, i.e., seeking support in religion, and lacking of humor (8 items; sample item: “I pray more than usual”; Cronbach’s alpha of .80). The COPE-NVI demonstrated good psychometric properties in previous studies on Italian samples, showing high internal consistency and test-retest reliability (Foà et al., 2015; Sica et al., 2008).

#### Sexting Behaviors

Five items were adapted from the Sexting Behaviors Questionnaire (Morelli et al., 2016), in order to measure the frequency of three sexting behaviors during COVID-19 lockdown (i.e., “Please report the frequency of your sexting behaviors during the current period of lockdown for the COVID-19 health emergency”). The *sexts* were defined as: “sexually suggestive or provocative text messages/images/videos shared via new technologies” (Chalfen, 2009). The assessed dimensions were: experimental sexting, i.e., engaging in sexting for exploring sexuality and intimacy in a relationship of trust (2 items: “how often have you sent sexts depicting yourself?”; “how often have you sent sexts to your dating partner?”); risky sexting, i.e., sexting in highly dangerous conditions (2 items: “how often have you sent sexts while using alcohol, marijuana or other drugs?”; “how often have you sent sexts to strangers or people exclusively met online?”); emotional sexting, i.e., engaging in sexting for regulating negative emotions (1 item: “how often have you sent sexts when you were bored, lonely, or sad?”). As in the original SBQ, all items were rated on a 5-point Likert-type scale from 1 (*never*) to 5 (*always or almost daily*). The items extracted from the SBQ have proven efficacy in measuring sexting behaviors in young people, showing good psychometric properties in different studies on Italian samples (Bianchi et al., 2019a; Morelli et al., 2017, 2020).

### Data Analysis

Data analyses were performed with the statistical program SPSS version 24.0. Preliminary analyses indicated that risky and emotional sexting were highly positively skewed, as usually observed for problematic behaviors (Marengo et al., 2019). Thus, these variables were log-transformed prior to perform data analyses, in order to approximate their distribution to normality. First, a multivariate analysis of variance (MANOVA) was run to compare the three relationship groups (not in couple; non-distance relationship; long-distance relationship) on the three sexting behaviors, entered as dependent variables. Correlations about all study variables were also computed. Subsequently, three hierarchical regression analyses were conducted to investigate the predictors of each sexting behavior (experimental, risky, and emotional) during lockdown. In the first step of each regression, individual variables (biological sex; age; sexual orientation), relational variables (having a dating partner vs. not; being in a long-distance relationship vs. not), and pandemic-related stress were entered as predictors. In the second step, coping strategies were added to the regression equation. Finally, three multiple mediation models were tested using the PROCESS macro for SPSS version 3.0, model 4, in order to verify which coping dimensions mediate the relationship from pandemic-related stress to each sexting behavior during lockdown. Based on results of the regression analyses, only coping strategies which significantly predicted each sexting behavior were entered as possible mediators in the corresponding model. The effects of individual and relational variables were also controlled for in each model. The indirect effects and their 95% confidence intervals (CIs) were estimated using bootstrapping with 5.000 samples (Preacher & Kelley, 2011). CIs are considered significant when they do not include zero (Hayes, 2017).

## Results

### Descriptive Analyses

During the COVID-19 lockdown, 664 participants (34.4%) reported to have engaged in experimental sexting behaviors at least once (obtaining mean scores > 1), while risky sexting was reported by 191 (9.9%, with mean scores > 1), and emotional sexting was reported by 274 (14.2%, mean scores > 1). Subsequent data analyses were then run on the whole sample. The MANOVA showed an overall significant effect of relationship status on sexting behaviors, *Wilk’s lambda* = .86, *F*(6, 3848) = 50.53, *p* < .001, *η*^*2*^_*partial*_ = .07, and significant differences emerged on each sexting behavior (see Table 1). Tukey’s *b* post hoc comparisons indicated that experimental sexting was significantly more reported by participants who were in a long-distance relationship during lockdown (vs. other groups), while risky sexting was significantly more reported by participants who were not in couple (vs. other groups); emotional sexting was instead significantly less reported by participants who were in a non-distance relationship (vs. other groups). Statistics are reported in Table 1. Bivariate Pearson’s correlations are reported in Table 2.

**Table 1:** Descriptive statistics divided by relationship status groups

		Relationship groups						
		Not-in-couple (*n* = 780; 40.4%)		Non-distance relationship (*n* = 322; 16.7%)		Long-distance relationship (*n* = 817; 42.3%)		
	Range	*M*	*SD*	*M*	*SD*	*M*	*SD*	*F*(df)
Experimental sexting	1–5	1.29 ^a^	0.66	1.21 ^a^	0.57	1.89 ^b^	1.11	*F*(2,1928) = 122.72, *p <* .001, *η*^2^_p_ = .11
Risky sexting*	1–5	1.13 ^c^	0.41	1.05 ^d^	0.21	1.09 ^d^	0.32	*F*(2,1928) = 6.87, *p <* .01, *η*^2^_p_ = .01
Emotional Sexting*	1–5	1.27 ^e^	0.71	1.07 ^f^	0.34	1.30 ^e^	0.77	*F*(2,1928) = 14.47, *p <* .001, *η*^2^_p_ = .02

Different letters indicate significant differences among groups: a < b; c > d; e > f

^*^Risky and emotional sexting were log-transformed before performing the analysis; however, non-transformed mean scores are reported in table for improving clarity

**Table 2:** Bivariate Pearson’s correlations and descriptive statistics on study variables

	*1*	*2*	*3*	*4*	*5*	*6*	*7*	*8*	*9*	*10*	*11*	*12*	*13*	*14*	*M*	*SD*
1. Biological sex	1														--	--
2. Age	−.05^*^	1													24.17	2.75
3. Sexual orientation	.01	−.07^**^	1												--	--
4. Dating relationship	.12^***^	.14^***^	−.06^*^	1											--	--
5. Long-distance relationship	.04	−.07^**^	−.05^*^	.71^***^	1										--	--
6. Pandemic-related stress	.15^***^	.02	−.03	.07^**^	.11^***^	1									3.50	1.00
7. COPE Social support	.23^***^	−.02	.08^***^	.08^***^	.08^**^	.14^***^	1								2.40	0.65
8. COPE Positive attitudes	.01	.01	0.02	.03	.01	−.10^***^	.16^***^	1							2.55	0.47
9. COPE Avoidance strategies	.03	−.04	.12^***^	−.04	−.03	.17^***^	.20^***^	.07^**^	1						1.59	0.36
10. COPE Problem solving	−.07^**^	.02	.01	.03	.02	−.07^**^	.33^***^	.44^***^	− .03	1					2.31	0.53
11. COPE Turning to religion	.19^***^	.05^*^	−.08^**^	.05^*^	.03	.07^**^	.07^**^	−.05^*^	−.15^***^	−.05^*^	1				2.39	0.52
12. Experimental sexting	.01	−.11^***^	.12^***^	.22^***^	.34^***^	.05	.10^***^	.01	.06^**^	.05^*^	−.10^***^	1			1.53	0.92
13. Risky sexting^a^	−.19^***^	−.03	.17^***^	−.07^**^	−.02	.02	.01	.02	.14^***^	−.02	−.10^***^	.30^***^	1		1.10	0.34
14. Emotional sexting^a^	−.09^***^	−.04	.11^***^	−.03	.06^**^	.09^***^	.06^***^	−.01	.15^***^	−.04	−.11^***^	.40^***^	.50^***^	1	1.25	0.69

Biological sex was coded as 0 = boys; 1 = girls. Sexual orientation was coded as 0 = heterosexual; 1 = LGB+. Dating relationship was coded as 0 = no; 1 = yes. Long-distance relationship was coded as 0 = no; 1 = yes

^a^Risky and emotional sexting were log-transformed before performing the analysis, but non -transformed mean scores are reported for improving clarity

^*^p < .05; **p < .01; ***p < .001

### Hierarchical Regression Analyses

The assumptions of hierarchical multiple regression analyses were preliminarily verified, with variance inflation factors falling within acceptable ranges. In the first regression analysis, conducted on experimental sexting, step 1 explained the 13.6% of the variance. Age had a negative significant effect (with higher scores for younger participants), whilst sexual orientation and long-distance relationship showed positive significant effects (with higher scores for LGB+ youths and for participants in a long-distance relationship). Step 2 added a significant 1.7% to the explained variance, showing a positive significant effect of social support, and a negative significant effect of turning to religion. The final model accounted for the 15.3% of the variance in experimental sexting.

The second regression analysis was performed on risky sexting behaviors. Step 1, explained the 7.1% of variance, detecting significant negative effects of biological sex and dating relationship (higher scores for men and not-in-couple participants), and significant positive effects of sexual orientation (higher scores for LGB+ youths) and pandemic-related stress. In step 2, adding a significant 1.7% to the explained variance, avoiding strategies showed a significant positive effect, whilst problem solving showed a significant negative association. This model explained the 8.8% of the variance in risky sexting.

In the third regression, performed on emotional sexting, step 1 explained the 4.4% of variance, detecting significant negative effects of biological sex and dating relationship (higher scores for men and not-in-couple participants), and significant positive effects of sexual orientation, long-distance relationship (higher scores for LGB+ youths and participants in long-distance relationship), and pandemic-related stress. Step 2 added a significant 2.6% to the explained variance, and detected significant positive effects for social support and avoidance strategies, and significant negative associations for problem solving and turning to religion. The final model explained the 7% of variance in emotional sexting (see Table 3).

**Table 3:** Hierarchical regression analyses on sexting behaviors

	Experimental sexting				Risky sexting				Emotional sexting			
	Step 1		Step 2		Step 1		Step 2		Step 1		Step 2	
Predictors	*R*^2^	*Beta*	*ΔR*^2^	*Beta*	*R*^2^	*Beta*	*ΔR*^2^	*Beta*	*R*^2^	*Beta*	*ΔR*^2^	*Beta*
	.13^***^		.02^***^		.07^***^		.02^***^		.04^***^		.03^***^	
Biological sex		−.02		−.01		−.19^***^		−.20^***^		−.10^***^		−.10^***^
Age		−.07^***^		−.07^**^		−.01		−.008		−.01		−.001
Sexual orientation		.13^***^		.12^***^		.17^***^		.15^***^		.12^***^		.09^***^
Dating relationship		−.006		−.006		−.07^*^		−.06^*^		−.12^**^		−.12^**^
Long-distance relationship		.34^***^		.34^***^		.03		.04		.14^***^		.14^***^
Pandemic-related stress		.02		.006		.06^*^		.04		.11^***^		.08^***^
COPE Social support				.07^**^				.04				.07^**^
COPE Positive attitudes				−.02				.04				.002
COPE Avoidance strategies				.03				.10^***^				.10^***^
COPE Problem solving				.02				−.06^*^				−.07^**^
COPE Turning to religion				−.10^***^				−.04				−.08^***^
Total *R*^2^	.15^***^				.09^***^				.07^***^			

Biological sex was coded as 0 = boys; 1 = girls. Sexual orientation was coded as 0 = heterosexual; 1 = LGB+. Dating relationship was coded as 0 = no; 1 = yes. Long-distance relationship was coded as 0 = no; 1 = yes

^*^p <.05; **p < .01; ***p < .001

### Mediation Models

**Fig. 1:**
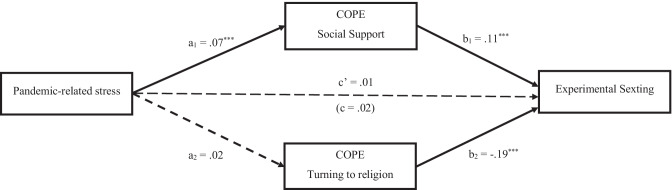
Mediating effects of coping between pandemic-related stress and experimental sexting. Notes: *a =* effects of pandemic-related stress on the mediators; *b* = effects of mediators on experimental sexting; *c*’ = direct effect of pandemic-related stress on experimental sexting; *c* = total effect of pandemic-related stress on experimental sexting. *p <.05; **p < .01; ***p < .001

The first mediation model was tested on the relationship between pandemic-related stress and experimental sexting. The total effect of pandemic-related stress on experimental sexting was nonsignificant, but the mediation model was tested anyway, in line with our hypotheses. The possibility to test indirect effects even in presence of a nonsignificant total effect has reached large consensus in research (e.g., Hayes, 2009). On the basis of results from the first regression analysis, only social support and turning to religion were settled as possible mediators in the hypothesized model. Pandemic-related stress emerged to be a significant positive predictor of social support, while its effect on turning to religion was nonsignificant. Social support in turn was a positive significant predictor of experimental sexting, while turning to religion was a significant negative predictor. Controlling for the mediators and the covariates, also the direct effect of pandemic-related stress on experimental sexting appeared to be not significant. However, a significant indirect effect emerged from pandemic-related stress to experimental sexting via social support, *a*_1_*b*_1_ = .01, *SE* = .003, 95% CI [0.003, 0.014], while the indirect path via turning to religion was nonsignificant, *a*_2_*b*_2_= − .004, *SE* = .003, 95% CI [− 0.010, 0.001]. Model’s statistics are reported in Fig. 1.

**Fig. 2:**
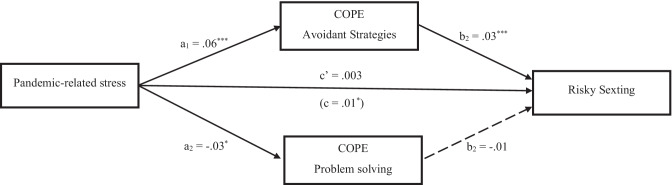
Mediating effects of coping between pandemic-related stress and risky sexting. Notes: *a =* effects of pandemic-related stress on the mediators; *b* = effects of mediators on risky sexting; *c*’ = direct effect of pandemic-related stress on risky sexting; *c* = total effect of pandemic-related stress on risky sexting. *p <.05; **p < .01; ***p < .001

The second mediation model was tested on the relationship between pandemic-related stress and risky sexting. Observing the results from the second regression analysis, only avoidance strategies and problem solving were settled as possible mediators. Pandemic-related stress showed a positive significant association with avoidance strategies, and a negative significant association with problem solving. Avoidance strategies in turn positively predicted risky sexting, whilst the effect of problem solving was nonsignificant. Controlling for the mediators and covariates, the direct effect of pandemic-related stress on risky sexting appeared to be not significant too. Only the indirect effect from pandemic-related stress to risky sexting via avoidance strategies turned out to be significant, *a*_2_*b*_2_ = .02, *SE* = .005, 95% CI [0.010, 0.031], while the indirect path via problem solving was not significant, *a*_2_*b*_2_ =.002, *SE* = .002, 95% CI [− 0.001, 0.006]. Model’s statistics are represented in Fig. 2.

**Fig. 3:**
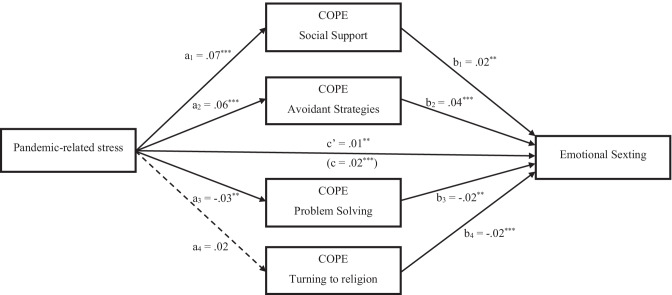
Mediating effects of coping between pandemic-related stress and emotional sexting. Notes: *a* = effects of pandemic-related stress on the mediators; *b* = effects of mediators on emotional sexting; *c*’ = direct effect of pandemic-related stress on emotional sexting; *c* = total effect of pandemic-related stress on emotional sexting. *p < .05; **p < .01; ***p < .001

The third mediation model was tested on the relationship between pandemic-related stress and emotional sexting. In the light of results from the third regression analysis, social support, avoidance strategies, problem solving, and turning to religion were settled as possible mediators. Pandemic-related stress was a significant positive predictor of social support and avoidance strategies, a significant negative predictor of problem solving, while its effect on turning to religion was nonsignificant. Social support and avoidance strategies also significantly and positively predicted emotional sexting, whilst problem solving and turning to religion were significant negative predictors. Controlling for all mediators and covariates, the direct effect of pandemic-related stress on emotional sexting was significant. Moreover, three significant indirect paths were found from pandemic-related stress to emotional sexting, via social support, *a*_1_*b*_1_ = .01, *SE* = .003, 95% CI [0.002, 0.015], via avoidance strategies, *a*_2_*b*_2_ = .02, *SE* = .005, 95% CI [0.009, 0.028], and via problem solving, *a*_3_*b*_3_ = .004, *SE* = .002, 95% CI [0.001, 0.010]. Only the indirect path via turning to religion was nonsignificant, *a*_4_*b*_4_ = − .003, *SE* = .002, 95% CI [− 0.008, 0.001]. Model’s statistics are represented in Fig. 3.

## Discussion

This study investigated the use of experimental, risky, and emotional sexting behaviors among emerging adults during the COVID-19 lockdown, providing the first data about sexting in Italy during pandemic, and exploring the relationships with pandemic-related stress and different coping strategies. Recent studies indicated an increase in sexting during the lockdown (Gabster et al., 2021), suggesting that it could have had a role in managing pandemic difficulties. However, to our knowledge, this study is the first evidence about the role of sexting in facing pandemic-related stress via coping strategies.

Regarding descriptive findings (Q1), most participants (42.4%) found themselves in a long-distance relationship during the national lockdown (while only 13.2% were long-distance before lockdown). The long-distance group reported the highest frequency of experimental sexting, which probably was perceived as the only available means to maintain intimacy with their partners during home-confinement, as suggested by pre-pandemic evidence about sexting in long-distance couples (Van Ouytsel et al., 2020). Other participants (40.4%) were not-in-couple, and reported the highest frequency in risky sexting, indicating that during lockdown they looked for online sexual contacts with strangers, or made sexting while using alcohol and drugs. Pre-pandemic sexting outside a stable relationship was used to pursue excitement and thrill (Van Ouytsel et al., 2017) and, according to our findings, single youths were more likely to search for at-risk online sexual encounters during home-confinement, perhaps in substitution of traditional casual sex. A smaller percentage of participants (16.7%) had a non-distance relationship during lockdown, and this group reported the lowest level of emotional sexting, suggesting that spending home-confinement with the partner was a positive resource, providing emotional support and reducing the need for compensatory behaviors.

Regarding the findings of regression analyses, there were no gender differences in experimental sexting, while risky and emotional sexting were more reported by men (vs. women), confirming recent studies (Bianchi et al., 2021; Morelli et al., 2021). Age negatively predicted experimental sexting, suggesting that younger adults have been more favorable to sexting with trustful partners during home-confinement, confirming the peak of sexting in early adulthood (Bianchi et al., 2019b). Risky and emotional sexting did not vary by age because, as other problematic behaviors (Marengo et al., 2019), they may depend on individual traits rather than developmental factors. LGB+ participants (vs. heterosexuals) reported more sexting in all dimensions, confirming their vulnerability to risky sexual behaviors (Nappa et al., 2021), and suggesting that they might have used online communication more often during lockdown, to escape unsupportive family environments (Woznicki et al., 2020). Moreover, participants who were single reported more risky and emotional sexting—suggesting their emotional vulnerability during home-confinement, and their attempts to substitute casual sex with online encounters. People in a long-distance relationship reported more experimental and emotional sexting—indicating the emotional vulnerability of youths who faced lockdown far from their partners, and tried to overcome physical distance with online intimate communication.

Controlling for individual and relational differences, the three sexting behaviors were differently associated with pandemic-related stress and coping strategies (Q2), and specific mediation effects were found for each dimension (Q3). Experimental sexting during lockdown was not predicted by pandemic-related stress, but only by adaptive coping (i.e., higher social support) and lower turning to religion. In line with the interpretation of experimental sexting as a new normality (Döring, 2014), our findings suggest that it was used to elicit emotional closeness in intimate relationships, rather than to escape negative affect like other problem behaviors (Kardefelt-Winther, 2014). Regarding the mediation model, pandemic-related stress positively predicted social support, which in turn positively predicted experimental sexting. Emotion-oriented coping related to social support has proven to be an adaptive coping response to pandemic difficulties in recent research (Park et al., 2020). An interesting indirect relationship also emerged from pandemic-related stress to experimental sexting via social support, suggesting that, during COVID-19 lockdown, experimental sexting was the result of adaptive coping to contrast pandemic worries: young people might have used this intimate communication to cope with pandemic-related stress, by looking for affection and support in their intimate relationships.

Risky sexting during lockdown was directly associated with pandemic-related stress, as well as with maladaptive coping strategies, i.e., higher avoidance and lower problem-solving. The mediation model showed that pandemic-related stress positively predicted avoidant coping, while negatively predicted problem solving; in turn, only avoidant coping was positively associated to risky sexting. The theoretical model of Lazarus and Folkman (1984) suggested that, in presence of uncontrollable environmental stressors, effective coping is emotion-oriented rather than problem-focused. Our findings provide support for this model, showing that pandemic conditions—which may be considered largely beyond individual control—have lead people to adopt more avoidant strategies, while reduced problem solving behaviors. Moreover, a significant indirect path emerged from pandemic-related stress to risky sexting via avoidant strategies. Therefore, our findings suggest that risky sexting works very similar to other problematic online behaviors (Melodia et al., 2020): Young people engaged in risky sexting during lockdown in response to negative feelings elicited by pandemic condition, and these behaviors appeared to be maladaptive attempts to escape pandemic-related stress via avoidant coping. Moreover, young people with poor problem solving abilities may have turned to risky sexting to manage their difficulties—such as sexual abstinence and social isolation—in absence of safer solutions, but this pattern was not involved in the relationship of risky sexting with pandemic-related stress.

Finally, emotional sexting during lockdown was predicted by pandemic-related stress, by adaptive (i.e., higher social support) and maladaptive coping (i.e., higher avoidance, lower problem solving), as well as by lower turning to religion, showing a functioning akin to both experimental and risky sexting. The mediation model confirmed the positive association of pandemic related stress with social support and avoidance, the both of which were positively associated with emotional sexting. Also the negative association of pandemic related stress with problem solving was confirmed, and problem solving in turn resulted negatively related to emotional sexting. Moreover, social support, avoidance, and problem solving appeared to mediate the relationship between pandemic-related stress and emotional sexting. Literature about this form of sexting is still scanty (Bianchi et al., 2019a); however, our findings suggest that emotional sexting during lockdown was a response to distress due to pandemic. Emotional sexting might have been the outcome of adaptive coping, when it was the result of looking for social support to contrast pandemic-related stress, but it might also have a maladaptive facet, as the outcome of avoidant strategies to escape pandemic-related stress, or when it was enacted in absence of more adequate problem-focused solutions.

Regarding the effects of turning to religion, the tendency to find emotional support in religious practices is a coping strategy with either positive or negative outcomes (Pirutinsky et al., 2020). In our findings, religious coping was negatively associated with sexting, probably because religion discourages sexting as well as each sexual behavior not accepted by its precepts. Research on sexting and religiosity is still in its infancy, however the absence of mediation effects suggests that this coping strategy did not interact with the relationship between pandemic-related stress and sexting.

### Limitations and Implications

The first limitation of this study is the historical and cultural context: the research was conducted in Italy during the national lockdown due to COVID-19 outbreak, and our results cannot be generalized to different countries, where the experience of pandemic may have been very different. Another limitation is the adoption of exclusively self-report instruments, which can be affected by the social desirability bias. However, online anonymous surveys are considered one of the best methods to evaluate sensitive information, guaranteeing perception of privacy and improving participants’ honesty. Finally, our results are based on correlational data, and great caution should be made in interpreting them causally.

Despite these limits, our study may bring novelty in research, providing the first evidence for the coping role of sexting behaviors, and distinguishing in them both adaptive and maladaptive facets. Research and applicative implications may also stem from our findings. Our models should be applied in post-pandemic research to explore the coping function of sexting in varying environmental conditions, beyond the COVID-19 lockdown. Clinical implications encompass both prevention and treatment of negative sexting outcomes, suggesting the possibility to early screen individuals at-risk for a maladaptive use of sexting. Prevention and treatment on at-risk youths should then focus on raising awareness about own coping responses, and on strengthening a positive stress management. Our findings may also have implications for social policies and public health, suggesting the need of more effective sexual education programs aimed to prevent negative outcomes of risky and maladaptive sexting. These educational programs should be implemented at a national level, targeting young people in universities and schools, and should focus on adaptive and dysfunctional facets of sexting, sustaining the development of healthy coping strategies, and providing information for a safer use of Internet and of online sexual behaviors.

## Conclusions

The present study provides evidence about the coping function of sexting behaviors, specifically showing that emerging adults have used sexting to cope with pandemic-related stress, via either adaptive or maladaptive coping strategies. This is also one of the first studies providing data about the spread of sexting in Italian young people during the COVID-19 lockdown, demonstrating the role of the online dimension to overcome physical distancing and social isolation in the exceptional condition of home confinement. Our findings also contribute to a promising line of research about the different facets of sexting (Bianchi et al., 2021; Morelli et al., 2020, 2021), indicating that experimental sexting may be an outcome of adaptive coping processes to face environmental stress, while risky and emotional sexting can also result by maladaptive processes related to avoid negative emotional states and by limited problem-solving abilities.

## Data Availability

Data will be available by the first author upon reasonable request.
